# Value of immunohistochemistry in the diagnosis of malignant cervical lymph nodes

**DOI:** 10.5935/1808-8694.20130112

**Published:** 2015-10-08

**Authors:** Décio de Natale Caly, Abrão Rapoport, Otávio Alberto Curioni, Rogério Aparecido Dedivitis, Claudio Roberto Cernea, Lenine Garcia Brandão

**Affiliations:** a PhD in Pathology - AC Camargo Hospital. Pathologist - Heliopolis Hospital - São Paulo.; b Senior Associate Professor - Department of Surgery, Medical School - University of São Paulo. Technical Director of the Department of Health - Heliopolis Hospital, São Paulo and São José Hospital Surgeon at the RBBP, São Paulo.; c Senior Associate Professor - Department of Surgery - Lusiada Foundation - Santos. Head of the Department of Head and Neck Surgery and Otorhinolaryngology - Heliópolis Hospital - São Paulo and Surgeon - São José Hospital - RBBP, São Paulo.; d MD. Full Professor. Larynx Group Supervisor - Department of Head and Neck Surgery, University Hospital - Medical School - University of São Paulo.; e Associate Professor - Department of Head and Neck Surgery, Medical School - University of São Paulo.; f Full Professor - Department of Head and Neck Surgery, Medical School - University of São Paulo. Department of Head and Neck Surgery, Medical School - University of São Paulo.

**Keywords:** carcinoma, squamous cell, immunohistochemistry, lymphatic diseases, lymphoma

## Abstract

The cervical lymph nodes are relevant due to the diversity of clinical entities. The use of immunohistochemistry is a real method to elucidate the diagnosis of adenopathy, both primary and metastatic neoplasms.

**Objective:**

To assess the value of immunohistochemistry in the diagnosis of cervical lymph nodes malignancies.

**Method:**

Retrospective study of the database histopathological specimens from 2009 to 2011.

**Results:**

Out of 32 biopsies of cervical lymph nodes, in 16 (50%) the immunohistochemistry was employed, being 68.75% (11) in hematological neoplasms and 31.25% (5) in carcinomas. It was used in all cases of lymphoma.

**Conclusion:**

The immunohistochemistry was used in 50% of the biopsies of lymph nodes under suspicion of malignancy, being 31.25% in epithelial lesions and 68.75% in lymphoproliferative lesions.

## INTRODUCTION

Reports of neck lymphadenopathies are described worldwide as one of the most common forms of peripheral manifestations of this disorder. The etiologies for this group of lesions are the most diverse, ranging from reactive states, infections, autoimmune diseases, primary and secondary neoplasms^1^, ^2^. Because of its etiological diversity, there is an age range of onset that can reach from the pediatric to the elderly patient.

Clinical research is extremely important in order to reach a more accurate diagnosis and to help decide which are the cases that require further and more specific investigation. Histological analysis is central to the assessment of these lymphadenopathies when clinical and/or radiological criteria alone are unable to establish a precise diagnosis, and especially in highly suspicious for malignancy cases. Histopathological evaluation is essential to establish both the histological type and its classification and thus, it is an important factor to help conceive and execute treatment. The difficulties in the histopathological analysis of biopsy specimens exist because of diagnostic variety and the pathologist experience.

The analysis and histological classification of tumors gained a few decades ago an important complementary technique with the advent of immunohistochemistry, promoting, over the years, its incorporation into routine diagnostic methods, facilitated thanks to improved technology, including more efficient antigenic recovery protocols, improved markers, lower costs and easier use of paraffin material. With this technique we became more accurate in determining the histogenesis of malignant neoplastic lesions, especially in cases of poorly differentiated neoplasms, as well as for establishing and finding the primary site in cases of metastatic neoplasms. Poorly differentiated neoplasms are difficult to diagnose, and have divergent interexaminer diagnostic rates. The use of an accurate immunohistochemical panel is essential to differentiate epithelial and mesenchymal cell lines^3^.

Metastasis in neck lymph nodes from squamous cell carcinoma is a common situation in cases of patients already diagnosed and treated for cancer of the head and neck, and it may as well be the first overt clinical manifestation. In general, squamous cell carcinoma, even the metastatic one, does not pose major histopathological diagnostic difficulties; however, in their less differentiated forms, it may raise diagnostic questions when only hematoxylin and eosin dyes are utilized.

We must not forget that immunohistochemistry brought about a diagnostic and prognostic revolution in the evaluation of primary neoplasms of the hematopoietic system^4^. The classification and identification of lymphomas was positively impacted, and we started to add the method to daily diagnostic routine, which is presently a mandatory item in pathology reports.

We noticed a lack of reports which analyze the use of immunohistochemistry in the histopathological evaluation of lymph node biopsies of the head and neck. Thus, the revision of the present series aims to assess the need to use this technique to aid in routine diagnosis.

## METHOD

This is a retrospective study carried out in the Registry Files of the Cancer Hospital (RHC) of the institution, from August 2009 to December 2011, based on the pathological results, being approved by the respective Research Ethics Committee under protocol # 170 (attached).

The inclusion criteria were: diagnosed cases of lymph node biopsies recorded at the RHC and which pathological results were filed in electronic media at the RHC. The exclusion criteria were: cases in which there was a diagnosis of squamous cell carcinoma in any sub-site of the head and neck.

Descriptive analysis was performed through the use of absolute and relative frequencies.

## RESULTS

After a pathology study, we found 32 reported cases of lymph node biopsies for diagnostic purposes, no primary lesion diagnosed. Of the 32 cases, 53.13% (17) were in males and 48.88% (15) in females, aged 23-85 years, with a mean of 52 years.

The most frequent histological cell lines found were lymphomas and squamous cell carcinomas (Table 1). Of the 32 cases, 16 were subjected to immunohistochemistry, most of which revealed that it was lymphoma (Table 2).

**Table 1:** Distribution of histological cell lines (n = 32).

	n	%
Squamous cell carcinoma	9	28.13%
Adenocarcinoma	3	9.38%
Thyroid papilliferous carcinoma	2	6.25%
Acinic cell carcinoma	1	3.13%
Undifferentiated carcinoma	1	3.13%
Large B cells diffuse lymphoma	6	18.75%
Hodgkin's lymphoma (classic)	1	3.13%
Hodgkin's lymphoma (nodular sclerosis)	3	9.38%
Follicular lymphoma	1	3.13%
Lymphoid hyperplasias	4	12.5%
Hemangioma	1	3.13%

**Table 2:** Immunohistochemistry use according to histogenic cell lines.

	With immunohistochemistry (n)	%	Without immunohistochemistry (n)	%
Lymphomas	11	34.38	0	0
Carcinomas	5	15.63	11	34.38
Others	0	0	5	15.63
Total	16	50	16	50

Lymphomas accounted for 68.75% (11) of the cases submitted to immunohistochemistry, and carcinomas for 31.25%^5^. Among the lymphomas, the most common subtype was the diffuse large B cell lymphoma; and among carcinomas it was the adenocarcinoma (Table 3).

**Table 3:** Distribution of the number of cases submitted toimmunohistochemistry according to the histological cell line (n = 16).

	Number of cases submitted to immunohistochemistry	%
Squamous cell carcinoma	0	0
Adenocarcinoma	3	18.75
Thyroid papilliferous carcinoma	1	6.25
Acinic cells carcinoma	0	0
Undifferentiated carcinoma	1	6.25
Large B cells diffuse lymphoma	6	37.5
Hodgkin's lymphoma (classic)	1	6.25
Hodgkin's lymphoma (nodular sclerosis)	3	18.75
Follicular lymphoma	1	6.25
Lymphoid hyperplasia	0	0
Hemangioma	0	0

Among the markers used in the immunohistochemical battery, the most used were: CD3, CD20, CD30 and CD 15 (Table 4).

**Table 4:** Distribution of the use of markers.

Marker	Number of times utilized
34BE12	4
35BH11	4
CK7	5
CH20	3
CK5/6	3
AE1/AE3	2
CD3	13
CD5	3
CD15	8
CD20	13
CD23	2
CD30	9
CD45	1
BCl-2	6
Ki67	5
HMB45	1
Vimentin	1
Thyroglobulin	1

## DISCUSSION

Enlarged lymph nodes in the neck of suspected neoplasia represent constitute an important clinical condition that requires proper diagnosis and treatment. Squamous cell carcinomas of the head and neck have a high risk of metastasis to regional lymph nodes and the confirmation of lymph node involvement has a direct impact on rates of disease control, with marked reduction in survival curves, requiring a correct diagnosis^5^.

In this study, we found that, of the 32 cases of lymph node biopsies, nine corresponded to squamous cell carcinoma, and of these, none had to be subjected to immunohistochemistry for diagnostic confirmation or establishing histogenic cell line. This value confirms the condition that the squamous cell carcinomas are, in most cases, lesions which, in their well differentiated and moderately differentiated shapes, causes no doubts about their histopathological diagnosis. Still in this series, we had other lesions of epithelial histogenesis, and in seven cases, four were subjected to immunohistochemistry.

The literature does not have comparative studies regarding the use of immunohistochemistry in malignant neck lymphadenopathy; however, we found reports of evaluations considering only poorly differentiated lesions in lymph nodes, with agreement rates confirming the diagnosis of carcinoma, with values ranging from 27 to 54%^6^. The major problem remains in the diagnosis of undifferentiated tumors present in 7.6% of head and neck cancer cases; and in this series we found 3.1%^6^. Thus, immunohistochemistry remains an important diagnostic tool for cases of poorly differentiated carcinomas in order to define histogenic cell lines^7^.

However, in this study, we found massive use of immunohistochemistry in lymphomas, accounting for 68.75% of its use. The classification of lymphomas in the past was only based on their morphological characteristics, evaluated by routine staining. However, as of the 80s, immunotyping techniques began to be used as a result of changing lymphoma diagnosis patterns^8^. Several classifications for lymphomas were based on new techniques and immunohistochemical markers that were emerging. Thus, we started having improved diagnostic approach to differentiate Hodgkin's lymphoma, and B and T non-Hodgkin ones, as well as better characterization of subtypes^9^.

Due to the great diversity in the clinical course of lymphomas, its proper classification noticeably changes its treatment, having an effective impact on survival rates. Thus, these days, it is practically unthinkable to have diagnostic conclusion without immunohistochemistry confirmation. In this series, all lymphomas were subjected to immunohistochemistry, the majority being diagnosed as large B cells lymphoma, followed by Hodgkin's lymphoma in the nodular sclerosis phase, a fact that coincides with the literature, in which the method was incorporated to all lymphoma cases^10^.

Still in this series, we found 12.5% of cases made up of reactive lymphoid hyperplasia and none was submitted to immunohistochemistry. The presence of benign lesions may bring about diagnostic questions and in such cases it may be necessary to use the method.

## CONCLUSION

Immunohistochemistry was used in 50% of lymph node biopsies of cases suspicious for malignancy, the use in epithelial cell line lesions occurred at 31.25% and 68.75% in hematopoietic cell lines, immunohistochemistry was used in 100% of the lymphoma cases.
